# Extinction accounts: Extinction and survival of French Polynesian tree snails (Partulidae)

**DOI:** 10.1017/ext.2026.10024

**Published:** 2026-10-15

**Authors:** Justin Gerlach

**Affiliations:** Peterhouse, University of Cambridge, UK

**Keywords:** *Euglandina*, metapopulation, *Partula*, *Platydemus*, recovery

## Abstract

The extinction of the *Partula* tree snails of French Polynesia was one of the most rapid and extensive extinctions recorded, with 75% of the species extirpated within a decade, due exclusively to the introduction of the predatory rosy wolf snail *Euglandina* spp. The pattern of extinction varied geographically and some *Partula* species persisted for 1–2 years post-*Euglandina* introduction. Partulids were able to withstand anthropogenic mortality: historically adult snails were exploited for cultural use and probably suffered population fluctuations following habitat disruption. The introduction of *Euglandina* represented a different scale of mortality, affecting all age classes. Since 1995, relict populations of some *Partula* species have been discovered; these have relatively high reproductive rates, live in areas with low predation pressures or are very dispersed. Most populations are unstable, with frequent disappearances and reappearances. One population showed evidence of full recovery, with a high density and high proportion of adults, before dispersing. A metapopulation structure is inferred, which would facilitate *Partula* survival despite the ongoing presence of *Euglandina* and a second predator, the New Guinea flatworm *Platydemus manokwari.* Predation pressures are substantially reduced and now seem to be too low to eliminate relict populations. This offers hope for the re-establishment of Extinct in the Wild species.

## Graphical abstract

**Figure:**
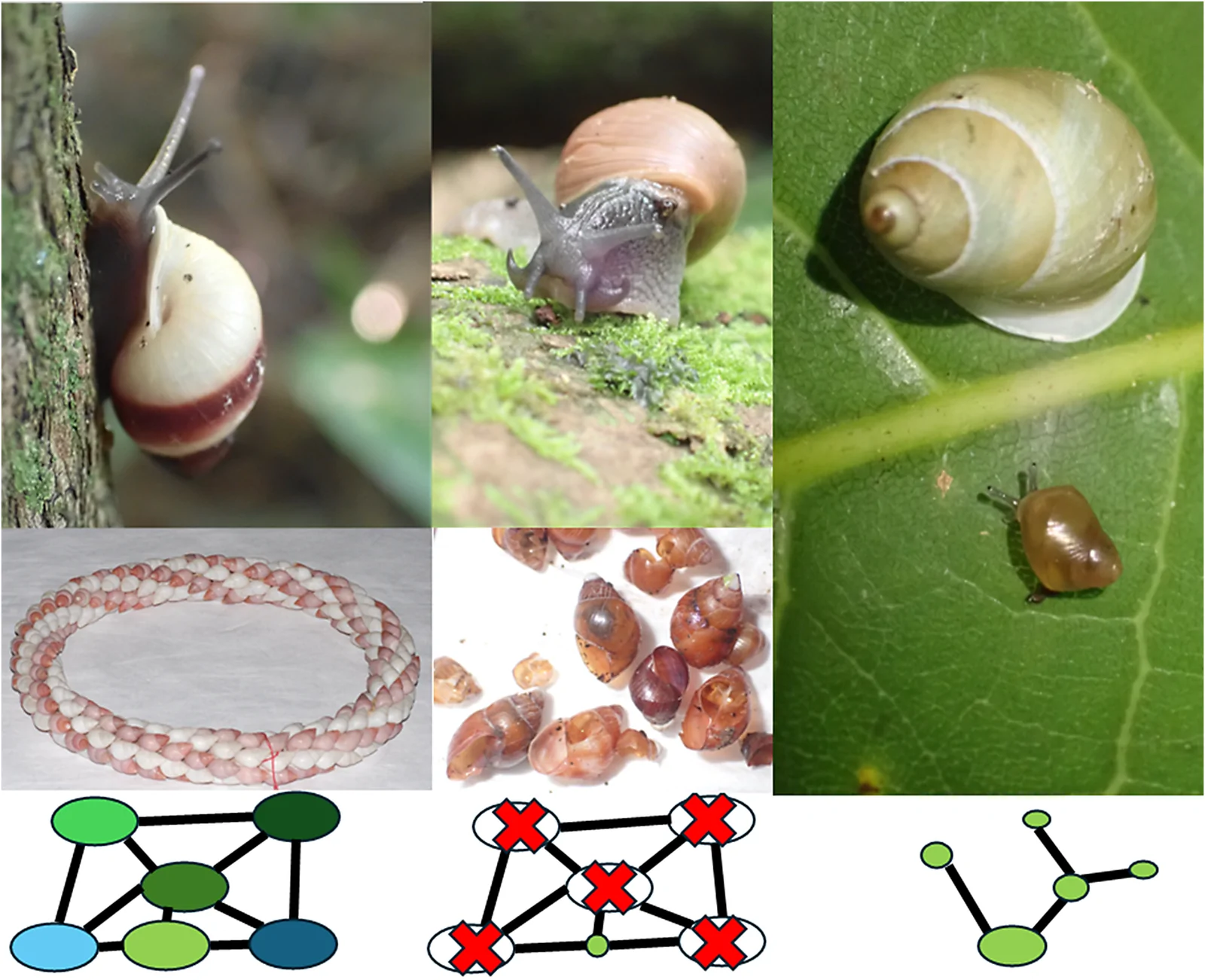

## Résumé

L’extinction des escargots arboricoles du genre *Partula* en Polynésie française fut l’une des plus rapides et des plus importantes jamais enregistrées, avec 75% des espèces disparues en une décennie, exclusivement à cause de l’introduction de l’escargot prédateur *Euglandina* spp. Le schéma d’extinction variait selon l’emplacement et certaines espèces de *Partula* ont persisté pendant un à deux ans. Les partulides ont pu résister à la mortalité d’origine anthropique: historiquement, les escargots adultes ont été exploités à des fins culturelles et ont probablement subi des fluctuations de population suite à la perturbation de leur habitat. L’introduction d’*Euglandina* a représenté une mortalité d’une tout autre ampleur, affectant toutes les classes d’âge. Depuis 1995, des populations relictuelles ont été découvertes; celles-ci présentent des taux de reproduction relativement élevés, vivent dans des zones à faible pression de prédation ou sont très dispersées. La plupart des populations sont instables, avec de fréquentes disparitions et réapparitions. Une population a montré des signes de rétablissement complet, avec une forte densité et une forte proportion d’adultes, avant de se disperser. Une structure métapopulationnelle est. proposée, qui favoriserait la survie de *Partula* malgré la présence continue d’*Euglandina* et d’un second prédateur, le ver plat de Nouvelle-Guinée *Platydemus manokwari.* La pression de prédation est. considérablement réduite et semble désormais trop faible pour éliminer les populations relictuelles. Ceci laisse entrevoir la possibilité d’un rétablissement de l’espèce disparue à l’état sauvage.

## Impact statements

Tree snails used to be found throughout the islands of the Pacific Ocean but most were driven to extinction by introduced predators in the late 20th century. The *Partula* tree snails species of French Polynesia were important in the development of the sciences of genetics and of cultural importance, their shells having been used to make necklaces and ‘crowns’ up until their extinction. They were also ecologically significant, feeding on decaying plant matter and on algae, fungi and bacteria growing on leaves. Since their extinction this vital ecological role has not been filled, and there has been a deterioration in forest health in that time. They were driven to extinction by the introduction of specialist snail predators in a failed attempt at biological control of an agricultural pest species. These predators caused exceptionally high mortality, to which *Partula* were not adapted. The process of extinction shows how quickly species can be lost and how vulnerable island ecosystems are to removal of such a key component. Some relict populations of Partula have been discovered and studying these has shown that some persist even though they remain vulnerable to predators. Since the extinction of most Partula populations the predators have also declined, and there now exists a new dynamic between predators and prey where the scattered distribution of Partula and their movement enables survival. Knowledge of this is being used to protect the existing populations and to re-establish some species that survive only in the conservation breeding programme. Both of these will contribute to restoring the health of the wider environment. The observation of the extinction process led to the *Partula* conservation programme. After four decades this now paying off, showing that conservation can work even when the causes of extinction cannot be completely eliminated or reversed.

## Lessons learned

- Historical harvesting of *Partula* snails for necklace-making represented an unnatural mortality factor but its focus on adults was ecologically less damaging than the indiscriminate mortality that followed the deliberate introduction of predators.
- Some partulid species survived either at geographical margins (high altitude or on headlands) where the slowing of predator range expansion resulted in lower predation pressure, or due to a combination of relatively high breeding rates and preferences for microhabitats (e.g. the tree canopy) that reduce predation pressure.
- The history of the relict populations shows that although bottlenecks inevitably reduce genetic variation, population recovery may still be possible if threat factors are reduced.
- Despite predator resurgences, partulids survive in metapopulations that are resilient to loss of subpopulations.
- Reintroduction programmes may succeed in creating coexistence between predator and their threatened prey by recreating metapopulation structures.

## Introduction

Current extinction rates may be at least 100 times higher than the background rate (Ceballos et al., 2015; Lamkin and Miller, 2016), with over 48,600 species assessed as threatened with extinction (IUCN, 2026). These threatened species are unevenly distributed due to a combination of taxon-specific threat factors, such as chytridiomycosis in amphibians or uneven taxonomic coverage (Edgar, 2025). In particular, relatively few invertebrate taxa have been monitored or assessed for extinction risk, resulting in under-recording of invertebrate extinctions (Régnier et al., 2015). For the majority of invertebrates, these ‘dark extinctions’ (Boehm and Cronk, 2021) are impossible to assess. However, molluscs are an exception as snail species leave identifiable shells, although the existence of extinct shell-less slugs can only be inferred indirectly (Gerlach et al., 2025a). Analyses of snail shell collections identify at least twice as many probable mollusc extinctions than have been recorded on the IUCN Red List of Threatened Species (Régnier et al., 2009). Island land snails appear to be at particular risk (Cowie et al., 2026) and the insular mollusc family with the highest proportion of extinct species is the Amastridae (94%) (Cowie et al., 2017), with an extinction rate of 0.4% per decade (Régnier et al., 2015). However, the extinction of 52% of the Partulidae represents the most rapid extinction, having taken place within the space of 10 years as a direct consequence of the introduction of at least two species of predatory *Euglandina* snails in misguided biological control efforts (Clarke et al., 1984; Murray et al., 1988; Gerlach et al., 2021). Within the Society Islands of French Polynesia, the process that led to the extinction of wild populations of at least 75% of Partulidae species was recognised in its early stages (Clarke et al., 1984; Murray et al., 1988), providing both information on the process and the opportunity to intervene (Pearce-Kelly et al., 1997).

The Society Islands Partulidae were particularly notable as the tree snails of the genus *Partula* were culturally and scientifically important. They were used in the manufacture of necklaces and other adornments on several islands, with a trade in these items in the late 20th century (Gerlach and Mu-Liepmann, 2026). Scientific research on the snails started with Henry Crampton’s work between 1907 and 1947. He sampled on an enormous scale, collecting all the snails he found in almost all the valleys on each island (Crampton, 1928). This was followed by the genetic studies of Bryan Clarke and Jim Murray on Moorea island from 1962 until the extinction of most populations in the 1980s (Clarke et al., 1984). Collections were also made on Tahiti and Moorea in 1970 by Jack Burch and Yoshio Kondo (Lee et al., 2009). Although these were significant collections, they do not appear to have had an impact on *Partula* populations, which remained abundant until the introduction of *Euglandina* (Murray and Clarke, 1980; Murray et al., 1982, 1993; Gerlach, 2016).

**Figure 1.**
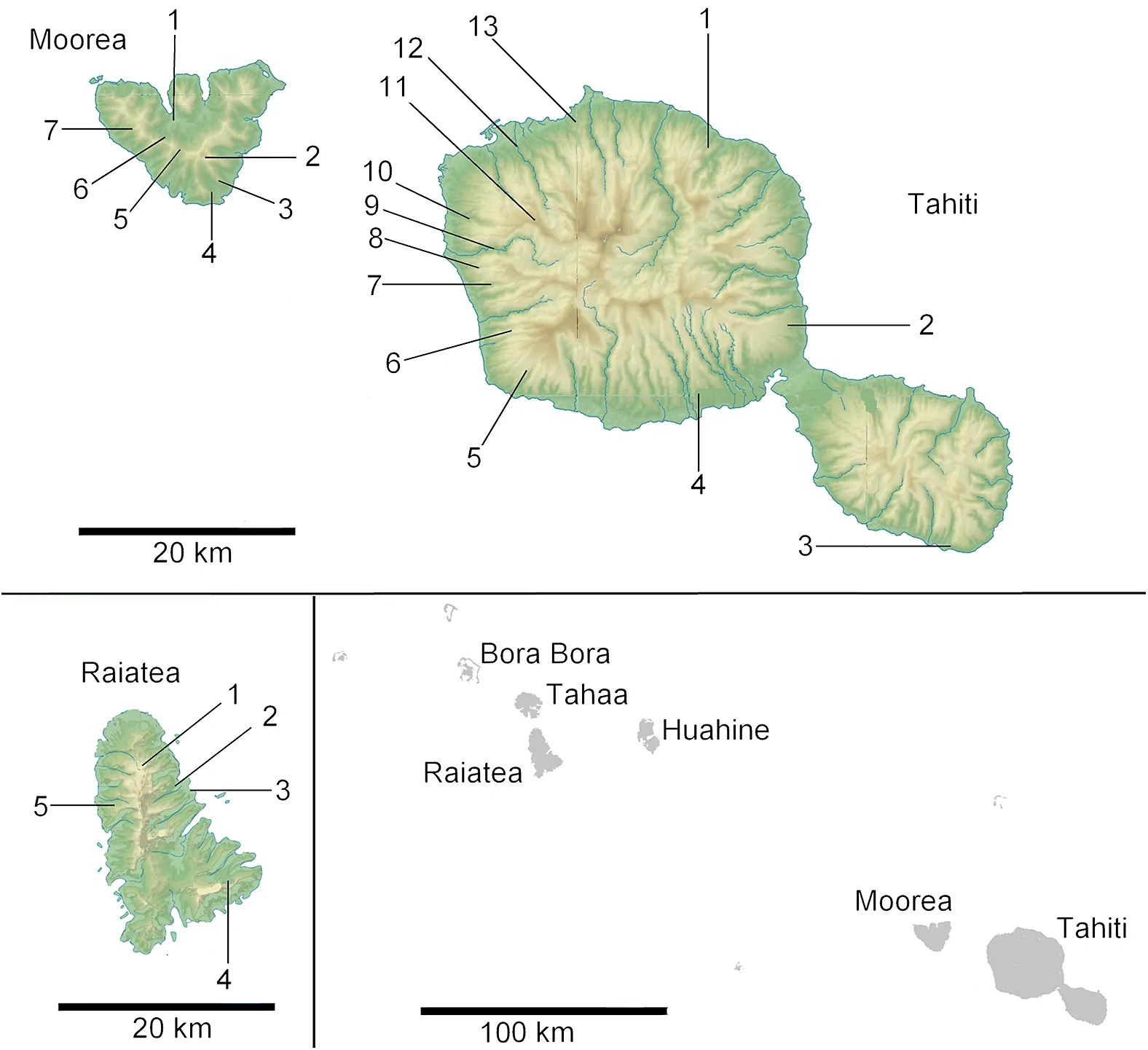
Society islands showing survey sites in the text. Moorea: 1 – Opunohu, 2 – Mt. Tohiea, 3 – Haumi, 4 – Maatea, 5 – Maramu-Vaianae, 6 – Morioahu, 7 – Moruu. Tahiti: 1 – Haapoponi, 2 – Vaitoare, 3 – Te Pari, 4 – Vaioo, 5 – Tereia, 6 – Vaipoe, 7 – Tiapa, 8 – Maruapo, 9 – Papehue, 10 – Matatia, 11 – Mt. Marau, 12 – Fautaua, 13 – Faarapa. Raiatea: 1 – Temehanirai; 2 – Hamoa; 3 – Avera; 4 – Hotopuu; 5 – Tevaitoa.

Following the first *Euglandina* introductions in 1974, partulid extinctions were documented on Tahiti and Moorea in the 1980s (Clarke et al., 1984; Murray et al., 1988) and on Huahine, Raiatea, Tahaa and Bora Bora in the 1990s (Pearce-Kelly and Clarke, 1992; Gerlach, 1994; Coote, 2001). Subsequently, a small number of relict populations of partulids have been found on Tahiti, Moorea, Raiatea and Huahine (Coote, 2007; Coote et al., 1999; Coote and Loève, 2003). From 2005 to 2020, Trevor Coote monitored the rediscovered populations (Coote, 2005–2020). He also identified the presence of a second invasive snail predator, the New Guinea flatworm *Platydemus manokwari* (Gerlach et al., 2021). Between 2020 and 2024, monitoring was carried out by Ravahere Taputuarai for the Direction de l’Environnement de la Polynésie française (Taputuarai et al., 2024). From 2003 the Partulid Global Species Management Programme has recorded wild partulids as part of post-release monitoring (Gerlach et al., 2023a, 2023b, 2024, 2025b). The data produced by different surveys are not directly comparable as not all were quantitative, and those that were quantitative were produced using different methods. These varied from single 20 × 20 m plots (Murray et al., 1982, 1993) to up to ten plots of 5 × 5 m (Gerlach, 1994, 2017; Coote, 2005–2020) and a combination of 2 m diameter plots and 2 × 20 m transects (Taputuarai et al., 2024). The data do, however, provide evidence of the presence of partulids and their predators. The locations of sites mentioned in the text are shown in Figure 1.

Here I review the disparate sources of data on the population declines, extinctions and survival, including the information on cultural exploitation and the scientific collections. Understanding species differences in the extinction process and evaluating the viability of surviving populations has implications for the *Partula* conservation programme which seeks to re-establish species that are currently extinct in the wild (Gerlach et al., 2025c).

## The biology of extinction

The Partulidae are tree snails found on islands throughout the Pacific Ocean: from the Marianas in the north-west to French Polynesia in the south-east. They comprise 114 species in three (Li et al., 2026) to five genera (Slapcinsky and Krauss, 2016). The most diverse genera are *Partula* (80 species) and *Samoana* (at least 21 species), and geographically the greatest diversity is in French Polynesia (Gerlach, 2016). There the Society Islands have 51 *Partula* species and four *Samoana*, the Marquesas Islands have eight *Samoana* and the low-lying Austral Islands are occupied by only two *Partula* species (one introduced). Within French Polynesia all but two *Partula* species are naturally single island endemics, whereas *Samoana* has seven single island endemics and five species found on between two and five islands (Gerlach, 2016).

Within French Polynesia, partulids were historically highly abundant, with densities recorded of up to 20 m^−2^ (Johnson et al., 1993). The great majority of partulids are arboreal species, with just four known to have been terrestrial, including two of the Society Island *Partula. Samoana* are all arboreal and tend to be associated with the canopy, whereas *Partula* are most abundant 1–3 m above ground (Murray et al., 1982).

The dietary ecology of most Society Islands species has been documented (Gerlach, 2016): they are mostly generalist consumers of plant epidermal tissue and detritus. The two terrestrial species have both been categorised as generalists. Most arboreal species are also generalists, but this group includes species that specialise on detritus caught in plant axils and on biofilm growing on leaves, especially fungi. Those at mid-heights also include some fungi specialists. In consuming biofilm, partulids were significant components in the ecosystem, removing organisms that grow over plant leaves. In addition, three Society Islands species have been documented to consume small snails as part of their diet (*Partula clara*, *P. hyalina* and *P. incrassa exigua*: Gerlach, 2016). At least in the case of *P. hyalina* this is deliberate killing and consumption of prey snails (pers. obs.).

With their generalist diets, most arboreal species are not closely associated with any particular host plant species. Exceptions are *Partula mirabilis, P. mooreana* and *P. tohiveana* which are associated with the climbing *Freycinetia* spp., but these are not exclusive associations. The latter two species are also restricted to higher elevation sites, presumably for climatic reasons. *Partula taeniata* favours shrubs and *P. suturalis* favours *Hibiscus tiliaceus* trees, but these are only tendencies (Murray et al., 1982). Because of this lack of strong association with any particular plant host, and their mostly generalist diets, partulids have been relatively tolerant of habitat invasion by introduced plant species.

Other aspects of their biology do make partulids more vulnerable to extinction. Almost since their first description, partulids have been known to give birth to live young (Férussac, 1821). As a result, their fecundity is low, with the number of births per year varying between different species, from 4 to 41 (Gerlach, 2016). It has been assumed that this slow reproductive strategy has arisen from a lack of predators on the islands (Cowie, 1992): no natural predation on partulids has been documented. Their low reproductive rates mean that they have a limited ability to recover from significant mortality events (Mace and Balmford, 2000; Cardillo, 2003; Hutchings et al., 2012).

A low reproductive rate probably contributes to restricted genetic diversity (Larson, 2012). Measures of heterozygosity are notably low: 0.07–0.18 in wild populations (Johnson et al., 1977, 1986). These values are considerably lower than for the Hawaiian tree snail *Achatinella lila* (0.53: Price and Hadfield, 2014; Price et al., 2015), the continental *Otala lactea* (0.44: Elliott and Pierce, 1992) and the endangered Japanese *Mandarina* species (0.33–0.88: Nishida et al., 2022). This limited genetic diversity is likely to reduce adaptability and there is an association between low heterozygosity and extinction risk (Spielman et al., 2004).

## The process of extinction

### Exploitation: 19th and 20th centuries

For Partulidae, a generalist ecology and tolerance of their habitats being invaded by introduced plants mean that population decline leading to extinction is most likely to be driven by direct mortality factors. These may be driven by human exploitation (cultural and scientific collecting) and introduced predators.

There is no evidence of partulid cultural exploitation before the mid-19th century: none have been found in archaeological sites in the Society Islands (Kahn 2018; Kahn et al., 2014; Christensen et al., 2018), although in 2009 two *Samoana* (Traversat et al., 2024) were found in a 13th century midden in the Marquesas Islands (identified as *S. gonochila* from photographs provided by Gabrielle Traversat); outside of French Polynesia, two *Partula hyalina* shells have been found in a rockshelter in the Cook Islands dating from the 15th to18th centuries (Kirch et al., 1995, 2017). All of these are isolated, unworked shells, suggesting merely accidental inclusion in human-associated deposits. The 18th- and 19th-century descriptions, illustrations and collections of Society Islands clothing and ornaments document flower decorations, but the only mollusc items are seashell earrings referred to by Cook (1821) and Banks and Dalton Hooker (1896). One such earring is in the collection of the Cambridge University Museum of Archaeology and Anthropology (item D1914.7, described by Cook [1821]: ‘Their personal ornaments, besides flowers, are few; both sexes wear ear-rings, but they are placed only on one side: when we came they consisted of small pieces of shell, stone, berries, red peas, or some small pearls, three in a string’). In the Marquesas, though Cook (1821) referred to ‘necklaces and amulets made of shells, &c.’. In 1819, the laws directed by the London Missionary Society suppressed practices such as dances and associated ornamentation (Tagupa, 1974), but by the late 19th century hats were being decorated and ‘crowns’ of *Partula* shells were used as hatbands to secure hats in the wind and also worn as headdresses (Huguenin, 1902). The oldest known *Partula* artefact from the Society Islands is a crown of *P. hebe* shells in the Muséum Le Havre from 1880, although there are two *S. inflata* necklaces from the Marquesas Islands from 1845 (Gerlach and Mu-Liepmann, 2026). In 1892, shell necklaces were part of the costumes for a major dance festival held on Tahiti (Gerlach and Mu-Liepmann, 2026). It is notable that Andrew Garrett, who was resident in the Society Islands for many years, gave very detailed accounts of the snails, their habits and geography in 1884, but makes no mention of their use at that time (Garrett, 1884). This may suggest that any cultural use was not widespread. However, by 1901, *Partula* necklaces and crowns were commonly worn by women on Raiatea (Huguenin, 1902) and in 1904 the island was described as being famous for ‘shell-wreaths’ and necklaces, with three species recorded as being used: *P. dentifera, P. faba* and *P. hebe* (Wragge, 1906).

**Figure 2.**
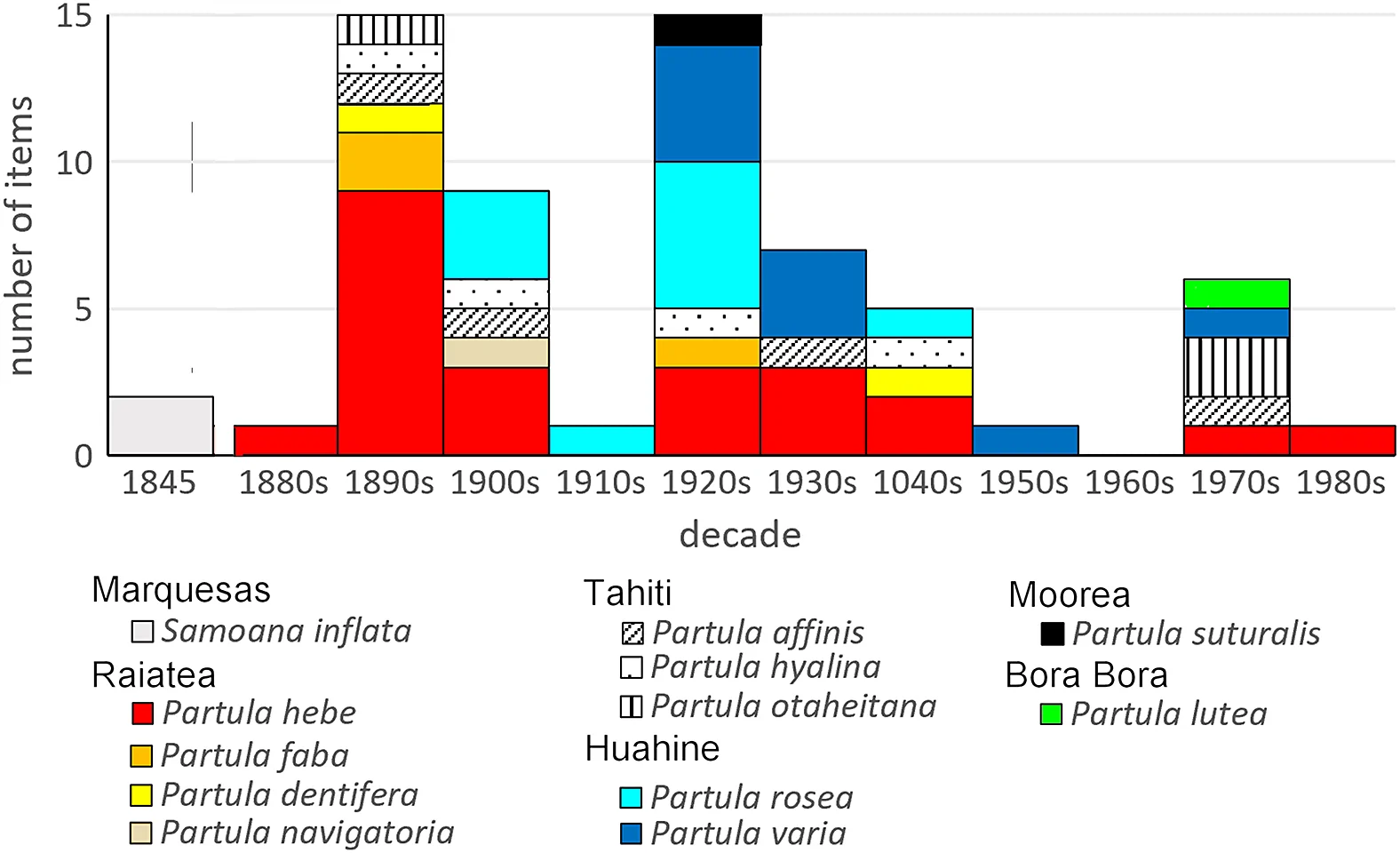
Numbers of artefacts containing French Polynesian partulid shells of different species from the study by Gerlach and Mu-Liepmann (2026). Original data in Supplementary Material.

A survey of partulid artefacts in the world’s museums (Gerlach and Mu-Liepmann, 2026) identified 70 Society Island items registered between 1880 and 1988. These comprise items from every island, and include 11 species. Most have shells of only one species, although six contain 2–3 species, including one which mixes two species from Huahine and Raiatea. Necklaces contain around 250 shells each and crowns 500, all live-collected adults (Huguenin, 1902; Gerlach and Mu-Liepmann, 2026). The scale of cultural exploitation cannot be accurately estimated; it appears to have been focussed on certain species: *P. hebe* of Raiatea makes up 43% of identified artefacts, and *P. rosea* and *P. varia* of Huahine make up 21% and 16%. In contrast, there seems to have been very little exploitation from Moorea, with a single item of *P. suturalis* out of the island’s five species (Figure 2).

The species composition also appears to vary over time (Figure 2): *P. hebe* is the most consistent, with items collected in every decade from 1880 to 1980, with a peak of seven items registered in 1891–1900. The second most abundant species, *P. rosea*, first appears in 1908 and *P. varia* in 1920. This might indicate a movement in the focus of cultural exploitation, from Raiatea to Huahine or it may be an accident of museum collection. Specimen notes support the latter; in the Bernice P. Bishop Museum, Hawai’i, a specimen of *P. dentifera* from Faarepa, Raiatea (BPBM 87282) was annotated by John W. Moore in 1926: ‘much used by native for making hat bands’, corroborating the observation made by Wragge (1906). However, only two surviving items using this species are known, one from 1897 (Musée de la Nouvelle-Calédonie: 98–4-15) and one from 1915 (Musée d’Aquitaine). This is a white to pale brown species, and is rarely banded, whereas the museum collections are dominated by necklaces of patterned or brightly coloured species (92%). Preservation of patterned items by collectors would account for the three most colourful species making up 69% of collected items.

By the 1930s, trade seems to have been substantial, at least on Huahine. In 1937, C.M. Cooke noted ‘Part. rosea is collected by the tens of thousands in Huahine and shipped to Tahiti for making “leis”’ (note on a necklace in the Academy of Natural Sciences, Philadelphia). If Cooke’s note is accurate, this could account for the observation that in the 1990s *Partula* were far less abundant on Huahine than on Tahiti or Moorea and had much less genetic diversity (Goodacre, 2002). The trade continued for a few years after the extinction of wild partulids, with necklaces being commonly sold until 1997. What were purported to be the last available artefacts were purchased in May 2001 (Coote and Loève, 2003), although one further seashell necklace containing some *P. rosea* was purchased on Huahine in March 2019 (Trevor Coote pers. comm.).

Cultural exploitation appears only to have been significant relatively recently; it may have had some local impact on populations, but based on the available evidence, it is unquantifiable. Scientific exploitation also began in the 18th and 19th centuries, initially on a small scale, but became significant with the arrival of Henry Crampton in 1906. He collected 400,000 partulids across all islands (Crampton, 1916, 1928, 1932; Gerlach, 2016). The geographic extent of the populations can only be tenuously determined from his field notebooks and, comparing his records with the situation in 1962, Jim Murray and Bryan Clarke (1980) were unable to identify any notable changes in distribution or relative abundance that could not be explained by differences in collecting localities. They collected around 100,000 *Partula* on Moorea in 1962–1968, removing all snails from their plots, with an estimated efficiency of 80% (Murray and Clarke, 1984). Their samples covered only a small proportion of the terrain and with total population density estimates of 11,200–15,000 per hectare (Murray et al., 1993) the collections would account for only a small proportion of the population. Several other collections were made in the 20th century, significant ones being the Mangareva Expedition of 1934 and collections made by Jack Burch and Yoshio Kondo in 1970–1 (Gerlach, 2016). Although these number into the thousands of specimens, nowhere do they exceed a hundred specimens from any one valley. Both cultural and scientific collecting may have represented significant mortality factors, but only on a very local geographic scale. In contrast, the predatory rosy wolf snail *Euglandina* and its mortality effects spread across all areas of the islands (Clarke et al., 1984).

### Extinction: 1982 onwards

At least two species were introduced to French Polynesia under the name *Euglandina rosea* (now known to be a complex of species: Meyer et al., 2017), first to Tahiti in 1974 in an attempt to control the invasive giant African snail *Lissachatina fulica.* The history of this failed attempt has been well documented elsewhere (Clarke et al., 1984; Murray et al., 1993; Gerlach et al., 2021). Key aspects are that *Euglandina* is a genus of specialised snail predators with a high reproductive rate and short generation time. They are voracious predators of most species of snail, hunting by following prey mucus on the ground and to several metres up trees. It has been calculated that one *Euglandina* will consume some 45–52 *Partula*-sized snails per year (Gerlach, 1994), far exceeding the reproductive potential of most *Partula.* The first introduction to Tahiti was followed by one to Moorea Island in 1977. Neither introduction was monitored and the first surveys of the predator’s distribution and impacts on partulids did not take place until 1980, on Moorea. At this point *Euglandina* was detected beyond the reintroduction point and a more extensive survey in 1982 found that it had spread widely. The only *Partula* found in invaded areas were small numbers (unspecified) of *P. exigua* (Clarke et al., 1984; Murray et al., 1993). In 1985 at least 50 *P. mooreana* were collected, along with some *P. mirabilis* and *P. suturalis.* In the next survey in 1986, the only surviving populations were in still uninvaded Haapiti valley in the extreme south of the island, where only two species were present: *P. suturalis* and *P. taeniata*; 6 months later these were extinct too (Murray et al., 1988). On Tahiti *P. diminuta, P. nodosa* and *P. producta* were collected in 1984 but in 1986 survivors of four species (*P. affinis*, *P. clara*, *P. hyalina* and *P. otaheitana*) were found in only two valleys (Murray et al., 1988). By 1991, all populations appeared to be extinct on both islands (Pearce-Kelly and Clarke, 1992).

*Euglandina* introduction to the other islands occurred later. Surveys in 1991 found partulids to be abundant on Huahine in the absence of predators and 246 were collected from two sites for conservation breeding (17 *P. arguta*, 8 *P. rosea* and 221 *P. varia*). By 1994 *Euglandina* was present and *Partula* had been almost completely eliminated; the date of complete extinction has been estimated at 1997 (Coote and Loève, 2003). In 1991 on Bora Bora, *Euglandina* was well established and partulids were only found as empty shells (Pearce-Kelly and Clarke, 1992). On Raiatea in 1991 *Euglandina* had largely eliminated partulids from several valleys but some survived in Hotopuu Valley where invasion was incomplete, and some valleys remained predator-free (Pearce-Kelly and Clarke, 1992). In Hotopuu, 9 *P. faba* and 1 *P. navigatoria* were found in the absence of *Euglandina*, and 9 live *P. hebe* remained where *Euglandina* was present, with abundant dead shells. Fewer predator-free valleys remained in 1992, although single live individuals of *P. faba* still survived in two invaded valleys (Gerlach, 1994); no other species were located alive. At this time *Euglandina* occurred at a density of 3,000 per hectare, and can be calculated to have consumed at least 45,450 *Partula* per year within that area (Gerlach, 1994). As the maximum pre-invasion *Partula* density was 36,667 per hectare, it would be expected that all arboreal *Partula* would be eliminated in less than a year. Terrestrial foraging by *Euglandina* is less efficient, giving a consumption of 12,840 *Partula* per year (Gerlach, 1994), still sufficient to eliminate the rarer ground-living *P. navigatoria* (4,000 per hectare). As a result of this high predation pressure, all partulids were concluded to be extinct in 1994 (Coote et al., 1999).

**Figure 3.**
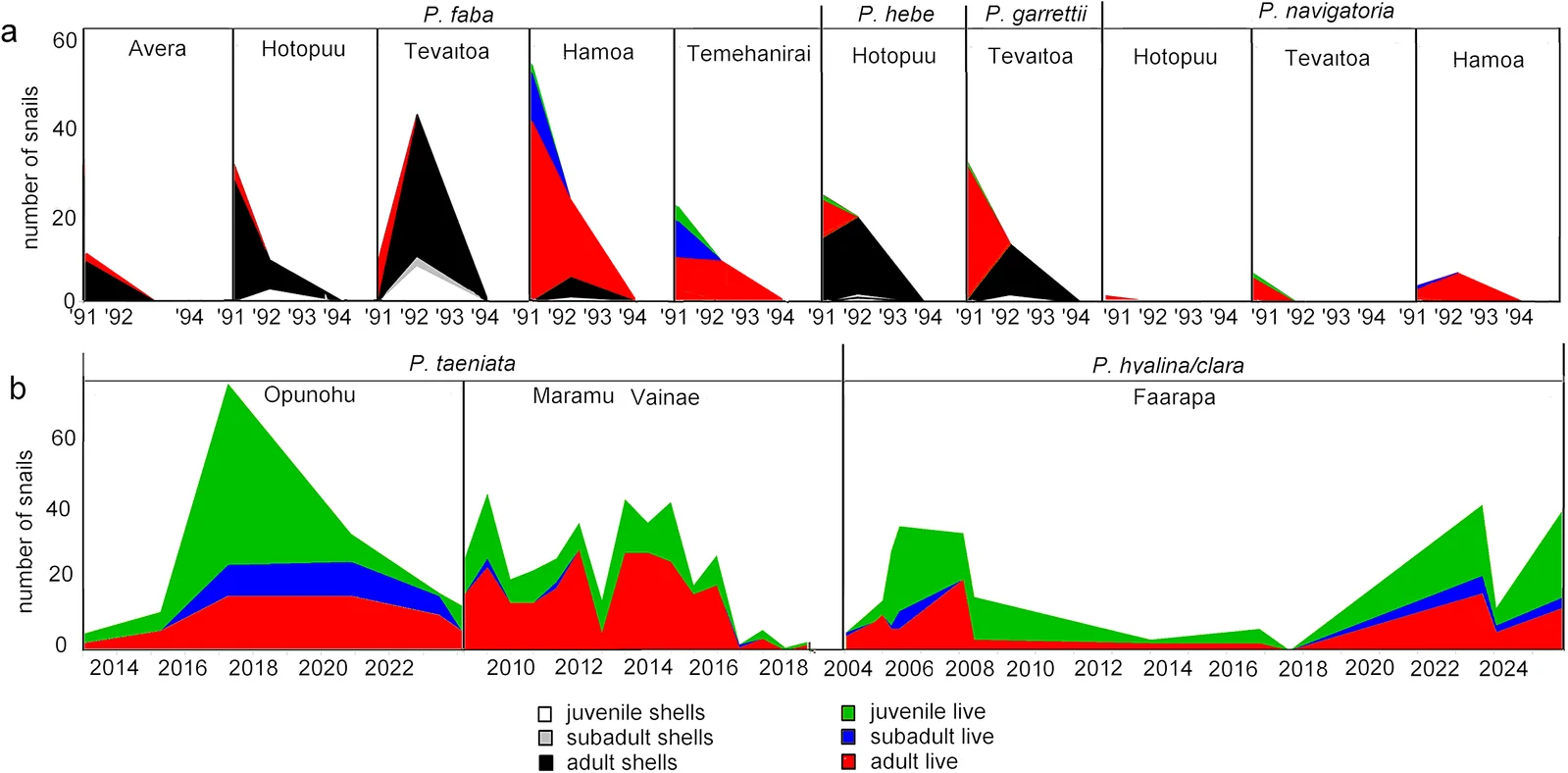
Age profile of *Partula* species in survey areas. a) the most abundant species on Raiatea in 1991–1994, valleys are in the order of invasion (Avera & Hotopuu in 1991; Tevaitoa in 1992; Hamoa & Temehanirai after 1992); b) monitoring data from Moorea (Opunohu and Maramu-Vaianae) and Tahiti (Faarapa).

Records of the numbers of live partulids found on Raiatea in 1991–4 are summarised in Figure 3. The surveys in these years involved manual searching for snails from ground level to 4 m above ground over a one-hour period. Search effort varied due to differing numbers of participants (four in 1991 and 1994, three in 1992) but exactly the same areas were visited on both occasions. The comparisons are therefore indicative but not fully comparable. On each occasion, all live and dead Partula located were identified to species and placed into age categories as juvenile (less than full adult size), subadult (full size but lacking an expanded shell lip at the mouth edge) or adult (full size with an expanded lip). The first sites to be invaded had fewest surviving partulids, and extinction was apparent within 2 years of invasion. This is clearest in the *P. faba* data whereas *P. hebe* and *P. garrettii* were only found alive in 1991 in the absence of *Euglandina*; in 1992 only abundant fresh shells were present. *Partula navigatoria* shows the same pattern of decline between 1992 and 1994. For all species, both recently killed and live populations had similar age profiles (Figure 3), indicating that predation was not size selective; all Raiatean partulids, except for the largest *P. faba* (Gerlach, 2016), fall within the size category of 4.5–25.0 mm shell length range where *Euglandina* predation is most efficient (Gerlach, 1994). The only snails to have been found in the presence of *Euglandina* were adult *P. faba.*

### Persistence and recovery: 1994 to the present

Since 1995, surviving *Partula* have been located at high altitude on Tahiti (*P. otaheitana* on seven peaks and *P. laevigata* on one), with isolated individuals seen on Moorea (*P. taeniata* on Mont Tohiea) and on Raiatea (*P. meyeri* on Mont Tefatua in 2006, with a fresh shell in 2024: Ravahere Tapuatarai pers. comm.). *Samoana* populations are also known in the upper parts of Tahiti and Huahine. These persist despite the occasional presence of small numbers of *Euglandina.* The south-east coastal area of Tahiti was not invaded by *Euglandina* until the 2000s and still supports three species at Te Pari.

Since 2004, scattered valley populations have been found: eight species in 35 valleys on Tahiti, and three in 11 valleys on Moorea (Table 1). In 2004–2005 monitoring plots were established in nine of the Tahiti valleys (Faarapa, Haapoponi, Maruapo, Matatia, Tereia, Tiapa, Vaioo, Vaipoe, Vaitoare) and four on Moorea (Haumi, Maatea, Morioahu, Moruu). These 5 × 5 m plots were located within identified partulid populations and varied in number according to the number of subpopulations located (Coote 2005–2020). Each plot was searched for partulids and predators for 30 min, monthly or annually depending on accessibility. There are also four sites on Tahiti where periodic surveys provide population trend data (Fautaua, Papehue, Te Pari and Mont Marau), and two on Moorea (Opunohu and Maramu-Vaianae). These sites have not been monitored systematically; surveys comprised teams of people (between two and six) recording all partulids found during a 30-min search.

**Table 1.** Wild partulid populations recorded since 2004 showing the most recent record of live animals Table 1. long description.

	sites	habitat type	monitored	*P. affinis*	*P. clara*	*P. compressa*	*P. hyalina*	*P. incrassa*	*P. laevigata*	*P. otaheitana*	*P. taeniata*	*S. attenuata*	*S. burchi*	*S. diaphana*
Tahiti	Ahonu	Forest	—		2012		2012					2012		
	Afererii	Forest	—				2008							
	Atehi	Forest	—		2020									
	Faaiti	Forest	—				2004							
	Faarapa	Forest	2005–2017		2024		2023					2023		
	Faaromai	Forest	—				(2026)							
	Faatautia	Forest	—				2023							
	Faurahi	Forest	—		2023		2008							
	Fautaua	Forest	—		2023		2025					2025		
	Fautaua – Faa Iti	Forest	—				2005							
	Hamuta	Forest	—									2022		2013
	Haapoponi	Forest	2005–2017		2026		2026					2023		
	Mahutaa	Forest	—											2024
	Maraeti’a	Forest	—										2022	
	Maruapo	Forest	2005–2019		2015									2022
	Matatia	Forest	2005–2023				2024							
	Nahoata	Forest	—				2023					2020		
	Onohea-Faarahi	Forest	—				2009							
	Onohea-Faaiti	Forest	—		2004		2004							
	Orofero	Forest	—		2021		2024							2002
	Papeava	Forest	—				2022					2022		
	Papehue	Forest	—		2026		2023					2023		
	Pihaiateta – Pito Hit	Forest	—							2024			2024	2023
	Puhi	Forest	—		2013									
	Rarouri	Forest	—				2021							
	Taapuna	Forest	—		2005									
	Tahaute-Tevaifaara	Forest	—				2005							
	Tehoroa	Forest	—				2007							
	Te Pari	Coastal forest	—	2024			2025			2024				
	Tereia	Forest	2007–2023		2023							2023		
	Tiapa	Forest	2004–2023					2022				2013		
	Tihiute	Forest	—				2012							
	Tipaerui-Iti	Forest	—				2020					2019		
	Tuauru	Forest	—				2006							
	Vaiaoa	Forest	—		2021							2021		
	Vaiiha	Forest	—		2004		2004							
	Vaioo	Forest	2007–2016				2015							
	Vaipoe	Forest	2007–2020		2020							2007		
	Vaipu	Forest	—				2008							
	Vaitoare	Forest	2005–2016				2021							
	Mont Aorai	Mist forest	—			2009				2023			(2025)	2025
	Mont Atara	Mist forest	—						2005	2005			2005	
	Mont Mahutaa	Mist forest	—											2024
	Mont Mapura	Mist forest	—											2023
	Mont Marau	Mist forest	—							2026			2013	2009
	Mont Pihaiateta	Mist forest	—							2024				2024
	Pic Vert	Mist forest	—											2002
Moorea	Afereaito	Forest	—									2026		2023
	Maatea	Forest	2005–2023								2013			
	Haumi	Forest	2007–2016								2009	2008		
	Maramu	Forest	—								2019			
	Morioahu	Forest	2009–2024								2012			
	Moruu	Forest	2005–2024								2024			
	Oio	Forest	—								2006			
	Oponohu	Mangrove	—								2026			
	Tefeo	Forest	—									2009		
	Vaianae	Forest	—								2015	2018		
	Mont Tohiea	Mist forest	—								2023			

*Note*: Dates in parentheses are for recent shells.

**Figure 4.**
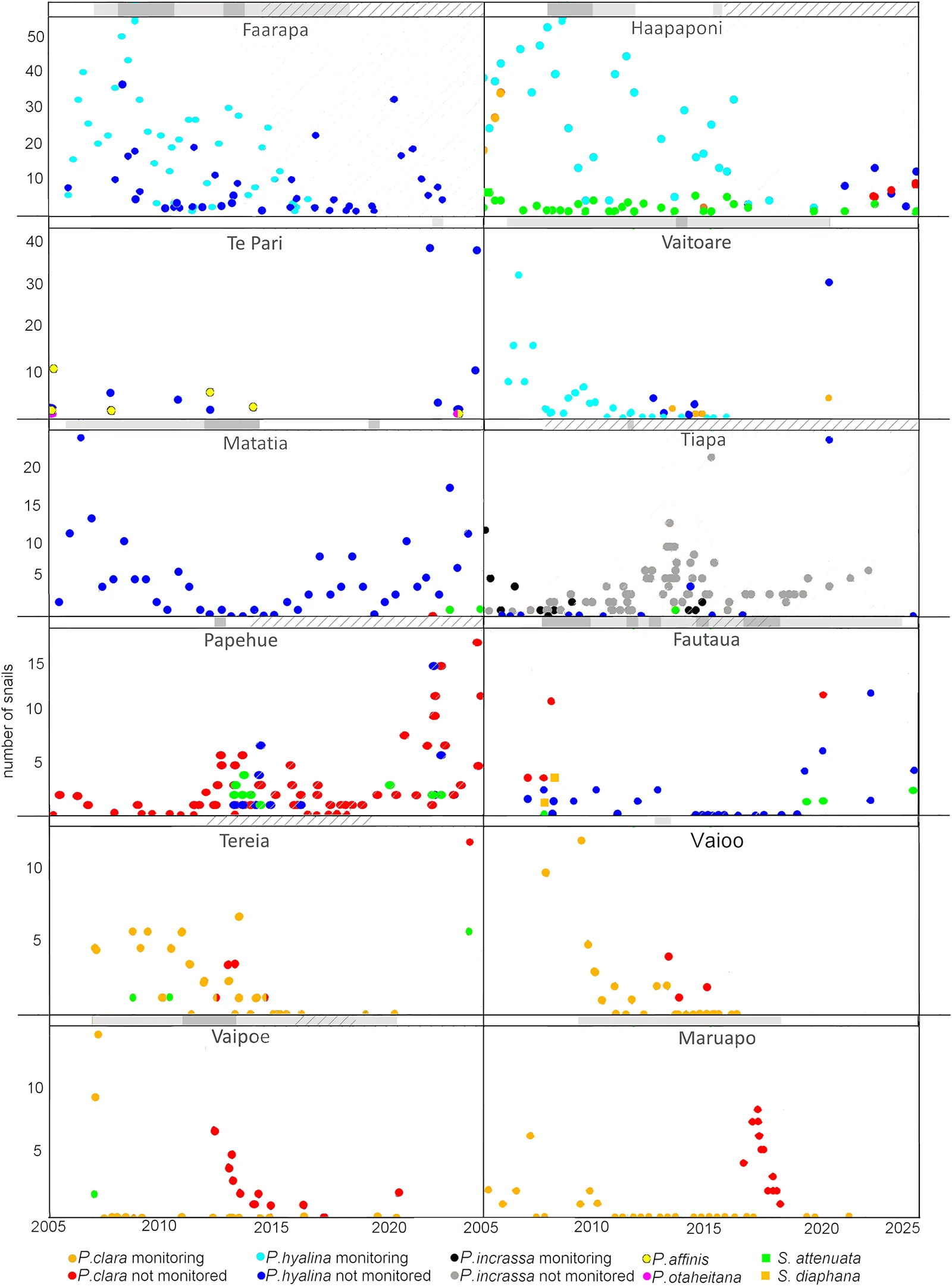
Monitoring data from Tahiti. The bar at the top of each graph shows predator presence: light shading – single live *Euglandina* found, dark shading – multiple live *Euglandina* present, hatching – *P. manokwari* presence. Data from (Coote, 2005–2020) and Taputuarai et al. (2024), available at https://doi.org/10.17863/CAM.133520.

**Figure 5.**
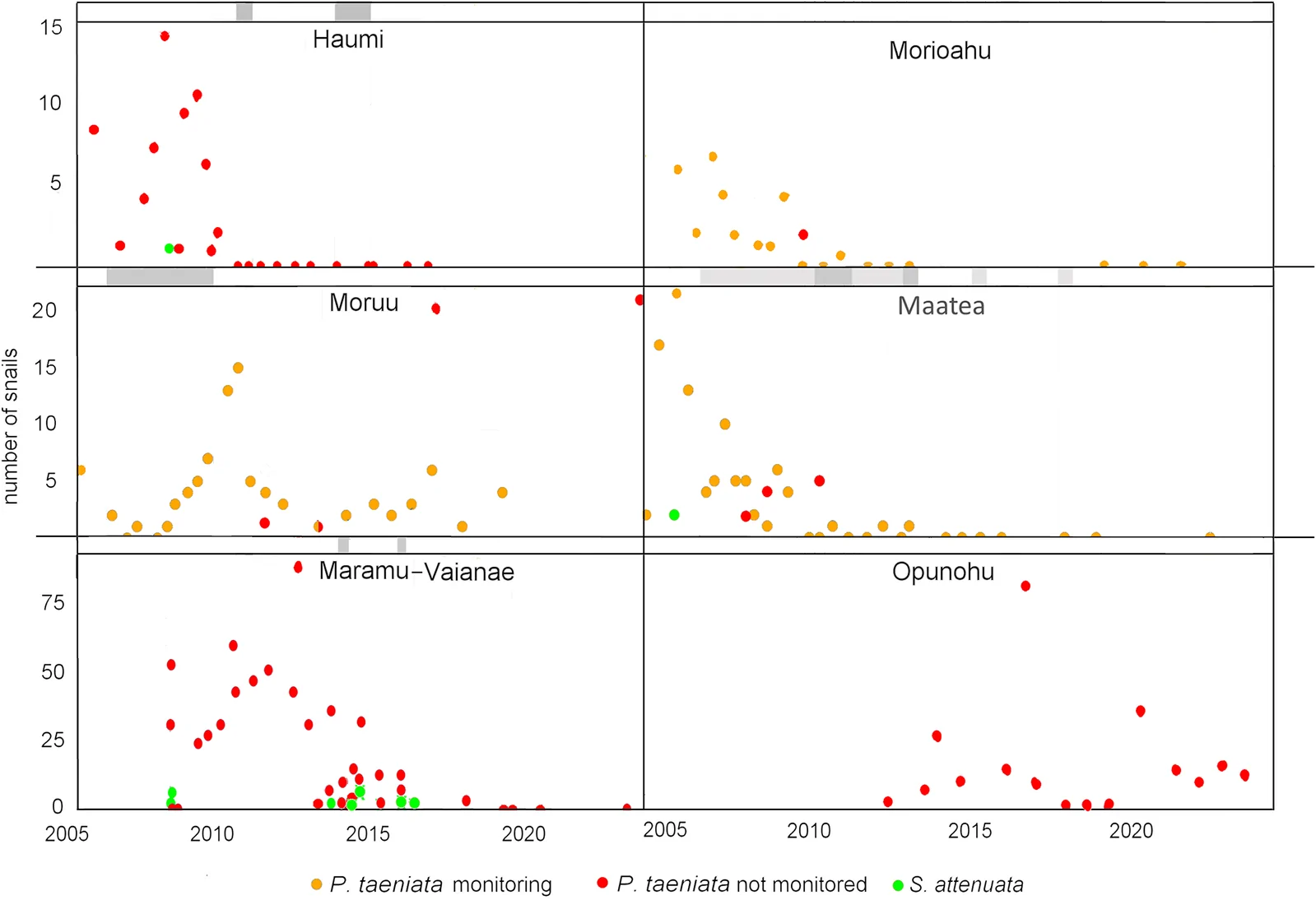
Monitoring data from Moorea. The bar at the top of each graph shows predator presence: light shading – single live *Euglandina* found, dark shading – multiple live *Euglandina* present, hatching – *P. manokwari* presence. Data from Coote (2005–2020) and Taputuarai et al. (2024), available at https://doi.org/10.17863/CAM.133520.

The monitoring data were analysed with a Generalised Linear Mixed Model using *Euglandina* numbers, *Platydemus* presence, mean mid-day temperature (°C) and monthly rainfall (mm) as predictors of partulid numbers. These were run separately for Tahiti and Moorea, for each partulid species and in combination. No significant associations were found between partulids and any combination of variables (Table 2). This is to be expected given that the data contain a high proportion of zero values, as many monitoring visits failed to locate any partulids, and predator abundance was not fully quantified. Despite these limitations, examination of the population trends and predator presence shows that there are no consistent patterns in monitored valleys (Figures 4 and 5), either in snails in monitoring plots or elsewhere in the same valleys (‘not monitored’ in Figures 4 and 5). Declines in the monitored populations corresponded to *Euglandina* resurgence in three sites (Maruapo, Matatia and Vaipoe), although additional *Partula* populations were found elsewhere in all of these valleys. Two declined with flatworm invasion (Tereia, and temporarily in Faarapa), but in two valleys partulids coexisted with abundant flatworms (Papehue and Vaipoe). In four valleys there were declines in the absence of changes in predator populations (Haumi, Maramu-Vaianae, Morioahu and Vaioo). One site has always been free of predators (Opunohu).

**Table 2.** Generalised Linear Mixed Model of partulid numbers Table 2. long description.

Source	Value	SE	df	t	*P*	95% interval	
						Lower	Upper
Intercept	19.955	24.128		0.827	1.000	−27.429	67.338
*Euglandina*	−1.433	2.961	607	−0.484	0.629	−7.248	4.383
*Platydemus*	−18.077	13.129	607	−1.377	0.169	−43.860	7.706
Temperature	−0.376	0.937	607	−0.402	0.688	−2.216	1.463
Rain	0.056	0.134	607	0.416	0.678	−0.208	0.320
*Euglandina* × *Platydemus*	0.948	0.612	607	1.549	0.122	−0.254	2.151
*Euglandina* × temperature	0.037	0.115	607	0.326	0.745	−0.188	0.263
*Euglandina* × rain	−0.001	0.001	607	−1.039	0.299	−0.004	0.001
*Platydemus* × temperature	0.643	0.502	607	1.281	0.201	−0.342	1.628
*Platydemus* × rain	0.013	0.007	607	1.854	0.064	−0.001	0.026
temperature × rain	−0.003	0.005	607	−0.600	0.549	−0.013	0.007

Data from Coote (2005–2020) and Taputuarai et al. (2024), available at https://doi.org/10.17863/CAM.133520. *Note*: Weather data from Meteo France (portail-*api.meteofrance.fr*).

Age profiles of monitored populations fluctuated markedly during monitoring periods (Figure 3). Except for Maramu-Vaianae, they had lower proportions of adults than was the case historically: 20.0%–68.8% adults compared to 70.7%–98.4% (Crampton, 1932; Murray et al., 1982). This difference may suggest that current populations are in an expansion phase, whereas the historic data come from stable, largely saturated populations. Most extant populations are very small, but three populations were large enough for comparison. On Tahiti, Faarapa monitoring plots were reinvaded by *Euglandina* in 2009, accompanied by many dead *Partula* shells and a drop in live adult numbers. This predation was insufficient to eliminate the population, which was ultimately lost to habitat clearance (Coote, 2005–2020). On Moorea, only two *Euglandina* have been seen near the Maramu-Vaianae population, with no evidence of any predation. This *Partula* population expanded, by 2012 reaching pre-invasion densities and a similar age profile (76.7%–79.1% adult). It then declined, and the adult proportion dropped to 32.0%. In the absence of any dead shells, this change can probably be attributed to dispersal, especially of adults; a population was found some 300 m away in 2026, which may be the result of this dispersal. Opunohu mangroves on Moorea have not been invaded by predators, but the *Partula* population has fluctuated in association with occurrences of habitat disruption. There, the adult proportion has also fluctuated, but remains relatively low: 20.0%–66.7%.

## Synthesis

Partulid populations have been assumed to be exceptionally vulnerable to predators having evolved in a predator-free environment (Groombridge, 1992; Gerlach, 1994; Bick et al., 2018), although the presence of extinct predatory birds has been hypothesised to explain the origin of shell polymorphisms (Johnson et al., 1993). Their vulnerability arises from having low reproductive rates and lacking anti-predator defences. Partulids faced other significant mortality factors in the past: the pollen and sedimentation record of Lake Temae, Moorea suggests that slash-and-burn agriculture was extensive in valleys and had destabilised hillsides around 950 BP (Stevenson et al., 2017). *Partula*-specific mortality factors were introduced with the advent of cultural exploitation in the 19th century. This may have reduced local populations; it has been speculated that similar exploitation of Hawaiian tree snails had population-level impacts (Hadfield, 1986; Cowie, 2022; Cowie et al., 2026), but it is impossible to assess the scale or impact of this exploitation in French Polynesia. However, the evidence suggests that *Partula* were able to withstand the removal of large numbers of adults from the population.

The introduction of *Euglandina* in 1974 represented a qualitatively different mortality factor, acting on both adults and juveniles. Even if *Partula* had faced bird predation in the past, *Euglandina* represented a different scale of pressure, occurring at extremely high densities and consuming all age categories. During the *Euglandina* invasion, all *Partula* species were vulnerable, but the speed of extinction varied. Small numbers of *P. exigua* and *P. faba* persisted for a few months after the extinction of all other species. These two differed markedly, the former being a small, partially carnivorous species high on vegetation (Clarke and Murray, 1969) whereas the latter was the largest Society Island partulid. *Partula exigua* may have survived briefly due to reduced predation on higher vegetation whereas isolated individuals of *P. faba* may have survived due to their relatively large size or by chance; this having been the most abundant Raiatean species (Gerlach, 2016).

*Euglandina* expansion was not uniform and it was predicted that it would fail to colonise high-elevation sites (Gerlach, 1994). The putative altitude ceiling at 1,000 m has proven to be incorrect, but partulid populations do persist above this altitude despite occasional *Euglandina* incursion. Monitoring data from Mont Marau (Coote, 2005–2020) demonstrate that *Partula otaheitana* survives these periodic small-scale invasions. Data from Mont Aorai are more limited but partulids continue to persist there too. On Raiatea, *P. meyeri’*s refuge on Mont Tefatua was assumed to be predator-free (Coote et al., 1999), however the collections made by J.W. Moore on the summit in 1927 included species which are now absent and it is probable that *Euglandina* invaded the summit temporarily, leaving a relict population of *P. meyeri* (Gerlach, 2016). A similar situation is found in coastal Te Pari where *Euglandina* did not arrive until 2001 (Coote, 2007) but it remains extremely scarce and *Partula* survive. At high elevations and on Tahiti’s south-eastern peninsula it is likely that the slow invasion by a diminishing wave of predators never caused the mass mortality seen in other areas. A similar pattern of slowing invasion has been reported on Chichijima Island in the Ogasawara group (Chiba and Cowie, 2016).

*Samoana* species are more resistant to *Euglandina* presence than *Partula* and survivors were found as early as 1995 (Coote et al., 1999). These have always been relatively scarce: Crampton collected only eight individuals from high elevations (Crampton and Cooke, 1953). Clarke and Murray (1969) noted that *Samoana* and *P. exigua* ‘share a tendency to rest higher than *taeniata* on the branches and leaves of shrubs and trees’, echoing Garrett (1884) on *S. attenuata*: ‘Owing to its peculiar habit of living on the foliage near the top of trees, it easily escapes observation’. This habitat preference may have reduced predation pressure on *P. exigua*, allowing it to persist for a further year, whilst predation on the *Samoana* species was further reduced by their more sparse distribution. Today the *Samoana* species are increasing in abundance on Tahiti and Moorea, even in valleys and low on vegetation (Gerlach, 2023a). This change in habits could be a result of competitive release following the decline of *Partula* species, or of some undetected environmental change.

*Partula clara*, *P. hyalina, P. incrassa* and *P. taeniata* have also been found to persist in some valleys (Coote et al., 1999; Coote, 2007; Coote and Loève, 2003). Bick et al. (2021) proposed that *P. hyalina* survives due to its association with ‘solar refuges’: edge habitat with high solar exposure, avoided by *Euglandina.* Whilst this may be a contributing factor, it does not apply to the other species and not all *P. hyalina* are found in such sites. It has also been hypothesised that the surviving species tolerate predation due to having relatively high reproductive rates (Bick et al., 2016, 2018). There is some support for this from captive populations where the species that coexist with *Euglandina* have annual birth rates of 22.6 (*P. taeniata*), 40.1 (*P. clara*) and 45.5 (*P. hyalina*), compared to 10.0–24.9 for the extirpated species (Gerlach, 2016). Nevertheless, annual birth rates for species with populations surviving occasional invasion are relatively low: 16.4 for *P. affinis* and 9.0 for *P. otaheitana*, supporting the idea that these populations survive due to low predation pressure, rather than their reproductive rate. High fecundity has not been sufficient for survival in all cases; even though *P. suturalis* and *P. garrettii* have high values (24.8–24.9) they did not persist in the wild following predation by *Euglandina.* The high birth rate of *P. suturalis* may be offset by a longer age to maturity (545 days to first birth, compared to 298 for *P. taeniata*: Clarke and Murray, 1969). Data for *P. garrettii* are limited but suggest maturity at about 298 days (Dave Clarke pers. comm.). The failure of this species to survive *Euglandina* invasion may be due to increased vulnerability, having occurred near the base of trees (Garrett, 1884).

Surviving *Partula* have fluctuated or declined within monitoring plots; however, additional populations have appeared elsewhere in the same valleys. This is therefore not a simple pattern of decline. In Fautaua Valley, the few partulids that survived the arrival of *Euglandina* disappeared following a substantial predator resurgence in 2008 through to 2021. However, several partulid populations have reappeared in various parts of the valley. A similar pattern of local populations appearing and disappearing is seen in Te Pari, despite the absence of significant numbers of *Euglandina.* There *P. hyalina* has a clumped distribution: several individuals can be found within areas of approximately 20 m^2^, interspersed with apparently suitable habitat lacking any snails (Gerlach, 2025b). Very few of these isolated populations can be found on sequential visits. This suggests a metapopulation structure which would make them resistant to local increases in mortality, and a high degree of mobility. A clumped distribution of *P. hyalina* was noted by Crampton (1916) who described them as living in ‘secluded colonies’, but he did not record whether the same colonies could be located over multiple years. Although *P. taeniata* was distributed throughout Moorea it also exhibited an uneven distribution; a 200 m transect showed two areas of high density separated by 40 m of very low density (Clarke, 1968), with a neighbourhood size of 200 m^2^ (Murray and Clarke, 1984). A mark-recapture study did record *P. taeniata* as being extremely sedentary, on average moving just 2.80 m in 5 years, with a maximum record of 7.37 m (Murray and Clarke, 1968). However, only 5% of snails were located after 5 years, attributed to a mortality rate of 50% per year (Johnson et al., 1993). As any snails dispersing beyond the 10 × 10 m sample plot would not have been detected, this 50% loss represents a combination of mortality and dispersal. Thus, *Partula* may be considerably more mobile than has been assumed.

A further contributor to population decline and extinction is often genetic erosion (van Oosterhout et al., 2026). As numbers of breeding age adults decline, inbreeding will increase and reduce genetic diversity, with the resultant loss in evolutionary adaptability increasing extinction risk (O’Grady et al., 2004). Partulids appear to have inherently low heterozygosity (Johnson et al., 1977, 1986), with measured wild values from original populations around a quarter of those of other species, and less than half those of threatened snail species (e.g. *Achatinella lilae* and *Mandarina mandarina*: Price and Hadfield, 2014; Nishida et al., 2022). Whilst this should reduce adaptability and increase extinction risk (Spielman et al., 2004), it may also mean that they are adapted to this situation. Within the conservation breeding programme genetic diversity has eroded further and some species declines are suspected to be due to inbreeding (Gerlach, 2016) but this is speculative. Other species have robust populations despite having declined to crucially low levels, as low as one individual in the case of *Partula taeniata simulans.* One of the most successful species in the conservation programme is *P. tohiveana*, now successfully re-established in the wild, despite having passed through a population bottleneck of just four individuals (Gerlach et al., 2026). This shows that, in at least some species, adaptability has not been lost even though genetic erosion must have occurred. With an evolutionary history of chance colonisations of islands, probably by single individuals, and hence strong founder effects, it may be possible that partulids are relatively resilient to the predicted impacts of inbreeding.

The decline of partulids in French Polynesia was solely due to the introduction of *Euglandina.* The patterns of population decline suggest that in the initial *Euglandina* population explosion in 1980–1994 the predators fed non-selectively and eliminated almost all populations within 2 years of contact. The few partulids that survived due to a relatively protected ecology, higher fecundity or fortuitous geography have started to expand and, in some cases, form viable populations. Subsequent predator resurgences are accompanied by a high mortality of adult *Partula*, but these resurgences differ from the initial invasion in that they are on a smaller scale, both in abundance and extent, and in affecting adult *Partula* rather than juveniles. *Euglandina* hunt by following their prey’s mucus trails (Clifford et al., 2003; Davis-Berg, 2012; Holland et al., 2012) and by chance encounter. At low prey densities trail-following is likely to be more important than chance encounters, leading to higher mortality of adults than of juveniles due to their larger trails. Partulid populations may persist through the maturation of the surviving juveniles offsetting adult losses.

The recovery of four partulid species in Fautaua Valley indicates that their metapopulations are likely to survive the predation pressures of *Euglandina*, even with local predator resurgences. The appearance of *Platydemus manokwari* flatworms has been an additional concern (Gerlach et al., 2021) but has not restricted partulid population growth, despite being a significant pressure on reintroduced *Partula* (Gerlach et al., 2023a). During invasion it reaches 30,000 individuals per hectare and, even when stabilised, fluctuates seasonally between 595 and 2,000 per hectare (Gerlach et al., 2021, 2023a, 2023b). Their daily consumption rate of 0.056 g (Gerlach, 2019) equates to 21.3 *Partula*-sized snails per year in ideal conditions, but this predator is vulnerable to desiccation. As a result its activity is constrained to wet days and predation is reduced by 63% above ground; it may be insignificant above 2.5 m (Gerlach, 2019; Anderson et al., 2026). These figures would indicate that the peak predation pressure would be substantial, but so reduced above ground as to make elimination of arboreal *Partula* populations unlikely. However, terrestrial species, such as *P. navigatoria*, might only be able to persist at the very lowest flatworm population densities.

Of the 51 French Polynesian *Partula* species at least 39 did not survive the initial introduction of *Euglandina* (a further eight species are probably extinct but may survive in poorly surveyed areas). Of these, ten are in the conservation breeding programme and these Extinct in the Wild species are the focus of the reintroduction programme with one, *P. tohiveana*, being successfully re-established in 2025 (Gerlach et al., 2026). These extirpated species were probably more vulnerable to extinction than the surviving ones due to lower fecundity, a more uniform distribution or an association with a more vulnerable microhabitat. The analysis of population decline and recovery offers hope that more species can be successfully re-established in the wild by creating a series of metapopulations that could enable survival and recolonisation even in the continued presence of predators.

The extinction of most French Polynesian *Partula* is a classic example of the present extinction crisis. It reinforces several well-known lessons: the vulnerability of island populations; the problems of inappropriate biological control measures; the importance of biosecurity to prevent introductions; and the value of conservation breeding. Research since the extinction event has shown the importance of thorough surveys as relict populations may persist undetected long after apparent extinction. These relict populations and the conservation breeding programme demonstrate that even though critically low populations would be expected to suffer inbreeding depression and loss of viability, some species recovery is still possible.

## Supporting information

10.1017/ext.2026.10024.sm001 Gerlach supplementary material Gerlach supplementary material

## Data Availability

All data analysed in this study are published or in reports held by Zoological Society of London and the Direction de l’Environnement de la Polynésie française.

## Supplementary material

The supplementary material for this article can be found at http://doi.org/10.1017/ext.2026.10024.

## Inclusivity statement

*Partula* conservation and monitoring in French Polynesia is a partnership between the Partulid Global Species Management Programme and the Direction de l’Environnement de la Polynésie française. It involves local communities in the reintroduction and monitoring at both institutional and individual levels.

## Table 1. Long description

On Tahiti 40 valley and 7 mountains have supported partulid populations since 2004 and 10 valleys and 1 mountain on Moorea. 8 species of *Partula* and 3 species of *Samoana* have been found on those islands.

Navigate back to Table 1..

## Table 2. Long description

The Generalised Linear Mixed Model incorporating *Euglandina* and *Platydemus* presence and rainfall and temperature failed to show any significant association between these variables and numbers of live partulids encountered.

Navigate back to Table 2..
