# Supplementary material for: Extinction accounts: Extinction and survival of French Polynesian tree snails (Partulidae)
Source: Camb Prism Extinct. 2026 Oct 15;4:e17. doi: 10.1017/ext.2026.10024 (PMC13748989; doi:10.1017/ext.2026.10024)
Supplement: Peer review history

# Reviewer report: Review: Extinction accounts: Extinction and survival of French Polynesian tree snails (Partulidae) — R0/PR2

**DOI:** 10.1017/ext.2026.10024.pr2

Dear author and editor,

This is a highly important and comprehensive review of the watershed conservation programme for the Partulidae land snails of French Polynesia. Overall, I agree with most of the manuscript’s presentation. However, there are several key aspects that should be improved for reproducibility and methodological and statistical rigour:

Partula exploitation: [lines 206–210] Ideally, please include a bar graph showing the change in species composition over time for the collected items. Please include Supplementary data if data is unpublished.

Partulid surveys of 1991–1994:

• [lines 290-295] It is not clear whether the search effort was standardised across the survey years. Please explain the survey methodology and annual search effort. If search effort varies with year, explain why the Partula decline is due to Euglandina predation and not search effort variation. Alternatively, consider weighting/adjusting the number of snails to the annual search effort to make the data comparable over the years.

• [line 296-297] define the partulid age profile categories (mm) and preferred prey size range (mm).

• [Fig. 2] The whole Fig. 2 needs reworking for better clarity. For Fig, 2a, provide the year on the x-axis for each valley: 1991, 1992, 1993, 1994. Also, contain each valley within a box with x- and y-axes. The species name can be in a box on top and stretched across all relevant valleys. Remake Fig. 2b into a similar format too.

Partulid surveys of 2004 onwards:

• [lines 315-317] Elaborate: Were these sites surveyed with a consistent methodology 2004-2025?

• [lines 318-319] Figs. 3-4 only visualised the raw data and doesn’t represent correlation analyses. Formal correlation analyses are needed to validate this statement as well as other statements in the paragraph. But considering the multiple factors (Euglandina/flatworm presence), rainfall and temperature may have influenced population changes over time, this dataset should be analysed using Generalised Linear Mixed Models (GLMMs) with repeated measures. GLMMs are robust for determining main factors influencing population changes for each species and deal with incomplete data. To ensure reproducibility, please include the raw data as Supplementary Material.

• [lines 748-749, Table 1] Recommend adding a column on vegetation type for each site. Some sites are in unusual habitats e.g. Oponohu mangroves. Also consider including vegetation as one of the factors for GLMM analysis of species population trends.

• Clarify terminologies in Figs. 3–4 [Fig3, lines 45-46]:

○ What do ‘clara monitoring’ and ‘clara not monitored’ mean? Please explain in fig. caption and main text.

○ In Fig3 caption and main text, What is the difference between ‘Euglandina presence’ and ‘heavy Euglandina presence’? What is the survey method used?

○ In Figs3-4, what is ‘limited survey’ (vs. ‘full survey’)? Explain in main text and Fig. caption.

○ Why are Maramu and Vaianae data separate in Fig4 but combined everywhere else in the manuscript?

• Formatting Figs. 3-4:

○ The Partula species names are missing in Figs3-4 captions. Add “P.”

○ Fig3, the grey points for ‘incrassa not monitored’ is hard to see. Better to create a separate bar on top of each graph that shows the presence/absence of Euglandina and Platydemus over time separately from the graph for the number of snails over time.

Other minor comments are:

• [line 20] correction: “French Polynesia”

• [line 23] rephrase for clarity: “1-2 years post-Euglandina introduction”

• [line 128] remove ‘-’ sign from “20m-2”

• [lines 275-276] rephrase for clarity: “In 1991 on Bora Bora, Euglandina was well established and only partulid shells were found”

• [line 319-320] Please clarify what does “although other populations were found in all of these” mean?

• [line 326-327] Please provide some possible reasons why there is such a big difference between these two studies.

• [lines 378-379] Similar to findings in Chiba and Cowie 2016, Fig. 3: https://doi.org/10.1146/annurev-ecolsys-112414-054331

• [line 439] Please specify which threatened species. Cite references.

• [lines 627-633] recommend uploading unpublished reports in a permanent repository with a DOI (e.g. Figshare) for reusability.

• Please provide citations for sentences with missing citations [e.g. lines 122–124, 201–205]

My best wishes to the author and I look forward to the revised manuscript.

Kind regards,

Junn Kitt Foon

# Reviewer report: Review: Extinction accounts: Extinction and survival of French Polynesian tree snails (Partulidae) — R0/PR3

**DOI:** 10.1017/ext.2026.10024.pr3

**Authors:** Isabel T. Hyman

**Affiliations:** Australian Museum, Australia

This is a very detailed and thoroughly researched review of the extinction of the partulid snails of French Polynesia. The narrative was engaging and made for a fascinating (but saddening) read. The figures are of good quality and the conclusions drawn are supported by the evidence. I have some small corrections and suggestions, listed below, but apart from that I recommend the paper be accepted.

I have used the continuous line numbering instead of the per page line numbering for reference, because it better matches the lines of text.

Line 2: ‘un-natural’ should be written without a hyphen – ‘unnatural’

Line 4: the word ‘of’ is missing – should be ‘deliberate introduction of predators’.

Line 82: I suggest replacing ‘as’ with ‘because’.

Lines 102-103: the last two words of line 102 and the first three words of line 103 appear to be in a smaller font.

Line 115: ‘current’ should be ’currently’.

Line 117: since the next heading is ‘The process of extinction’, this one should be ‘The biology of extinction’ (add the word ‘the’).

Line 120-121: this sentence is not grammatically correct. I would replace it with ‘The most diverse genera are Partula…’

Line 135: you say ‘arboreal species tend to be more varied’ (compared to the two terrestrial species, which are both generalists). Do you mean that some arboreal species are generalists while others are specialists? However, later (e.g. line 142) it appears that most are still generalists. I think this just needs a little more clarity and consistency.

Line 142: insert a comma after ‘diets’.

Line 144: ‘where’ should be ‘which’.

Line 161: my reading of Nishida et al. suggests a range of observed heterozygosity of 0.38-0.88 for Mandarina mandarina.

Line 249: I would suggest that the first subheading at line 166 should simply read ‘Exploitation’ and that you should add a second subheading at line 249 called ‘Extinction’. This breaks up the text better, and it seems to fall into three sections more naturally. You could even put the date range for each subheading as you did for the third one.

Line 275-276: since only one island is mentioned, you do not need ‘on the former’.

Line 279: missing the word ‘of’ – should be ‘…in the absence of Euglandina…’.

Line 329: I suggest replacing the colon with a full stop and starting a new sentence.

Line 411: I suggest a semicolon instead of a colon after ‘plots’

Line 413-414: this sentence is not grammatically correct. I suggest removing the word ‘where’ and adding a comma: ‘In Fautaua valley, the few partulids that survived…’

Line 453: the word ‘of’ is misspelt as ‘oof’

# Editor report: Recommendation: Extinction accounts: Extinction and survival of French Polynesian tree snails (Partulidae) — R0/PR4

**DOI:** 10.1017/ext.2026.10024.pr4

**Affiliations:** Free University of Berlin, Germany

Dear Dr. Gerlach

I have now received two review reports for your manuscript, both of which are positive. However, they do request some minor revisions to your manuscript, so please prepare a ‘response to comments’ document so show how you have taken these comments into account when you make your revisions.

I note that Reviewer 1 requests the use of a GLMM, in response to your text regarding ‘correlations’. I am of the opinion that there may be some misunderstanding here, and that this analysis is unlikely to be necessary for this type of manuscript. However, if you do decide not to undertake such analysis, please be careful to justify your decision thoroughly, and to revise your manuscript accordingly to remove any unsupported claims regarding correlations.

I look forward to receiving your revised manuscript.

Best regards

Tom Evans

# Editor report: Decision: Extinction accounts: Extinction and survival of French Polynesian tree snails (Partulidae) — R0/PR5

**DOI:** 10.1017/ext.2026.10024.pr5

**Affiliations:** Durrell Institute of Conservation and Ecology, University of Kent, United Kingdom of Great Britain and Northern Ireland

No accompanying comment.

# Reviewer report: Review: Extinction accounts: Extinction and survival of French Polynesian tree snails (Partulidae) — R1/PR7

**DOI:** 10.1017/ext.2026.10024.pr7

Overall, the manuscript has been mostly revised to satisfactorily address reviewer comments. However, the GLMM results and discussion (lines 345-355; Table 2) indicated four major issues that must be resolved to fit the model (if possible).

(1) The current Gaussian/Linear GLMM produces uninterpretable results (e.g. 95% Confidence Interval for the intercept is -27.4). The dataset contains count data (Partulid numbers), which should follow a Poisson or Negative Binomial distribution. So, it should be better to refit the model as a Poisson GLMM or Negative Binomial GLMM with a log link function.

(2) The intercept estimate (Value = 19.955) has an enormous standard error (SE = 24.128). This means the model cannot reliably estimate the baseline snail count when temperature = 0 and rain = 0. Transform the continuous variables of temperature and rainfall through mean-centering each variable to reduce intercept instability and multicollinearity. This can be done by subtrating the mean. e.g.: temp_centered = temperature - mean(temperature)

rain_centered = rain - mean(rain)

(3) The model is over-parameterised. Among the 10 fixed-effect predictors (main effects + 2-way interactions), not a single term is statistically significant. High standard errors across predictors suggest that including all interaction terms simultaneously is inflating variance (multicollinearity). To fix this, start with a simpler model containing main effects only. Add interaction terms only if there is a specific prior hypothesis or clear evidence of improvement via Likelihood Ratio Tests (LRT) or Akaike Information Criterion (AIC).

(4) GLMMs require both fixed and random components. The output provided shows only fixed effects. Please ensure random intercepts (e.g. (1 | Site) or (1 | Plot)) are properly specified and check their variance estimates to verify that the model structure accounts for spatial or temporal grouping. This should reduce overdispersion.

# Reviewer report: Review: Extinction accounts: Extinction and survival of French Polynesian tree snails (Partulidae) — R1/PR8

**DOI:** 10.1017/ext.2026.10024.pr8

**Authors:** Isabel T. Hyman

**Affiliations:** Australian Museum, Australia

The author has responded to all queries thoroughly and the manuscript looks great. Two minor typographical errors (which can be corrected in proof stage): on page 12, line 481, there is an extra bracket. Also on page 6, line 173, ‘plant’ should be ‘plants’.

# Editor report: Recommendation: Extinction accounts: Extinction and survival of French Polynesian tree snails (Partulidae) — R1/PR9

**DOI:** 10.1017/ext.2026.10024.pr9

**Affiliations:** Free University of Berlin, Germany

Dear Dr. Gerlach

Thank you for your thorough revision. One reviewer has some further comments on your analysis which I would like you to address. Please produce a point by point response letter to describe how you have responded to these comments.

I also think it would be worth adding a short section on methods, to describe the process used to identify the literature and to analyse the monitoring data. The text describing the analysis is quite brief and feels like an afterthought in its current location - it should be expanded and included in a dedicated methods section.

# Editor report: Decision: Extinction accounts: Extinction and survival of French Polynesian tree snails (Partulidae) — R1/PR10

**DOI:** 10.1017/ext.2026.10024.pr10

**Affiliations:** Durrell Institute of Conservation and Ecology, University of Kent, United Kingdom of Great Britain and Northern Ireland

No accompanying comment.
